# Historische Anmerkungen zur Ethik in der Psychiatrie: Herausforderungen im 19. Jahrhundert

**DOI:** 10.1007/s00115-024-01657-x

**Published:** 2024-05-27

**Authors:** Heinz-Peter Schmiedebach

**Affiliations:** Institut für Geschichte und Ethik der Medizin, Universitätsklinikum Hamburg-Eppendorf, Martinstraße 52, 20246 Hamburg, Deutschland

**Keywords:** Psychiatrischer Paternalismus, Humanität, Menschenexperimente, „Conträre Sexualempfindung“, Degenerationslehre, Psychiatric paternalism, Humanitarianism, Human experiments, “Contrary sexual feeling”, Degeneration theory

## Abstract

Mit dem Entstehen einer frühen Psychiatrie um 1800 entstand eine Reihe von Fragen zum Umgang mit einer Personengruppe, deren „fremdes“, irritierendes und störendes Verhalten als Phänomen eines Krankseins betrachtet wurde. Im Kontext der wachsenden Bedeutung der Menschenrechte erhielt der Begriff der Humanität eine hohe Bedeutung als Referenz für die frühen Psychiater. Anhand historischer Quellen wird dargelegt, dass trotz vielfachen psychiatrischen Bekenntnisses zur Humanität bis in die erste Dekade des 20. Jahrhunderts die konkrete psychiatrische Praxis von patriarchalischen Ordnungsregimes beherrscht, später von den Herausforderungen der somatisch-physiologischen und experimentellen Forschung sowie biologistisch-rassistischen Vorstellungen überlagert war. Die damit verbundenen neuen ethischen Fragen wurden zwar teilweise innerhalb der Psychiatrie thematisiert, verhinderten aber nicht eine Zunahme der Bewertung der psychiatrischen Kranken als „minderwertig“.

## Abstract (en)

With the emergence of an early psychiatry around 1800, a number of questions arose on dealing with a group of persons whose “alien”, irritating and disruptive behavior was considered to be a phenomenon of being sick. In the context of the growing importance of human rights, the term humanitarianism attained a high relevance as the reference for early psychiatrists. Based on historical sources it is shown that despite a multitude of psychiatric beliefs on humanitarianism the established psychiatric practice was dominated by patriarchal order regimes up to the first decade of the twentieth century, later superimposed by the challenges of somatophysiological and experimental research as well as perceptions of biological racism. The associated new ethical questions were partially addressed within psychiatry but did not prevent an increase in the assessment of the mentally ill as “inferior”.

## Einführung

Im folgenden Beitrag werden verschiedene Aspekte zur Ethik in der Psychiatrie [6–10] in einen historischen Kontext gestellt. Hinsichtlich des Rahmens, der das Thema umgibt, ist darauf zu verweisen, dass es trotz der sich bereits im Corpus hippocraticum zu findenden medizinethischen Regeln, wie das Gebot des Nutzens und das Schweigegebot, voreilig wäre, davon auszugehen, dass es in der Antike und den späteren Jahrhunderten eine unter den Ärzten breit geführte kontinuierliche ethische Diskussion gegeben habe. Dazu liegen keine einschlägigen schriftlichen Quellen vor. Im 19. Jahrhundert sind verschiedene Ereignisse auszumachen, die eine solche innerärztliche Debatte in Deutschland in Gang setzten. Insbesondere ab den 1880er-Jahren erhielt die Diskussion einen Auftrieb durch die propagandistische Ausschlachtung von Menschenversuchen durch politische Bewegungen, die der modernen naturwissenschaftlichen Medizin ablehnend gegenüberstanden. Wenngleich auch in der Blütezeit der experimentellen Medizin des 19. Jahrhunderts moralische Kriterien für die Vornahme von Versuchen an Menschen existierten, war eine systematische Thematisierung nicht gegeben [3, S. 236]. Jedoch wurden aus verschiedenen Gründen in eben dieser Zeit politische Verordnungen erlassen, die erste konkrete Regelungen bezüglich der Durchführung von Menschenversuchen formulierten [19, S. 20–27].

Diese Veränderungen waren der Wirkmächtigkeit eines sich seit der Aufklärung entfaltenden philosophischen und politischen Diskurses geschuldet, bei dem es um die Rechte, die jedem einzelnen Menschen aufgrund seines Menschseins zugestanden werden sollten, und um Menschenwürde ging. Dabei spielte auch die sich im 19. Jahrhundert entfaltende moderne Öffentlichkeit mit ihren vielfältigen Facetten eine wesentliche Rolle. Diese Ideen wurden auch von einigen psychiatrisch tätigen Ärzten aufgegriffen. Auf diese Weise wirkten gesellschaftliche Auseinandersetzungen und philosophisch-politische Gedanken in die Medizin und in die Psychiatrie hinein. Ohne diese gesellschaftlichen Diskussionen und Veränderungen hätte es die medizinethischen Debatten nicht gegeben. Eine Betrachtung medizinethischer Überlegungen und ganz besonders diejenigen innerhalb der Psychiatrie ab 1800 ist ohne Bezug zu den historischen sozialen und politischen Diskussionen der Vergangenheit nicht möglich oder doch nur sehr begrenzt aussagekräftig. Gerade in Zeiten politischer und/oder sozialer Krisen wirkten diese gesellschaftlichen Debatten auf Medizin und Psychiatrie ein. Dies konnte auch eine Umkehrung bis dato geltender Werte bewirken. Aus Menschen, denen man zunächst mit Fürsorge begegnete, wurden „Ballastexistenzen“, denen im Interesse von Volk, Vaterland und Rasse keinerlei Existenzberechtigung mehr zugestanden wurde [1].

Die grundsätzliche Frage hinter all diesen Veränderungen war die folgende: Wie geht eine Gesellschaft mit Personen um, die in ihrem Benehmen in vielfältiger Weise auffällig geworden waren, andere Menschen irritierten und Alltagsabläufe störten. Das Verhalten dieser Personengruppe manifestierte sich als ein „Fremdsein“. In den modernen europäischen Gesellschaften wuchs einer Expertengruppe die Aufgabe zu, den Charakter dieses Anderen, des Fremden zu erforschen und Wege zu finden, um damit, entsprechend den gesellschaftlichen Normen, umzugehen. Diese Experten gehörten dem Ärztestand an, weil man die infrage stehenden Phänomene spätestens seit dem Ende des 18. Jahrhunderts, teilweise auch schon früher, als Ausdruck eines Krankseins und damit auch tendenziell als heilbar betrachtete [2, S. 245]. Daraus ergab sich, dass auch professionspolitische Beweggründe in dieser ethischen Debatte einen gewissen Einfluss hatten. Mit dem Blick auf die Profession kommt dann auch das Selbstverständnis der Psychiater in den Blick und zwar in dreifacher Weise: im Verhältnis der Profession zur Gesellschaft, zweitens in dem Verhältnis zu den anderen medizinischen Disziplinen und drittens zu den Patienten und Patientinnen. Damit sind auch Fragen von Bedeutung wie: Was waren die Interessen der Profession? Verstehen sich Psychiater als Vertreter von Gesellschaft, Volk, Nation, Rasse oder mehr als Interessenvertreter der Patienten und Patientinnen? Dabei konnte es zu deutlichen Zielkonflikten kommen. Die sich entfaltende medizinische Disziplin Psychiatrie war mit einem dreifachen Gestaltungsfeld ausgestattet. Sie verfolgte erstens ordnungspolitische Funktionen, zweitens besaß sie einen medizinischen Charakter und drittens schließlich stellten sich im Hinblick auf die sich im psychiatrischen Feld befindenden Personen auch unterstützende Aufgaben. Dieses Dreieck war keineswegs immer in einer gleichschenkligen Weise gestaltet. Es gab Zeiten, in denen die ordnungspolitischen Aufgaben das professionelle Feld stark dominierten. Seit einigen Jahrzehnten nimmt die Frage der professionellen Gestaltung einer unterstützenden Funktion für die betroffenen Personen an Bedeutung zu.

Mit diesen Darlegungen ist der Rahmen für die folgenden Ausführungen abgesteckt. Es geht um die historische Kontextualisierung von im weitesten Sinne der Psychiatrie zuzurechnenden Überlegungen und Handlungen, die das Verhältnis zu psychisch und sozial auffälligen Menschen betreffen und die sich im Spannungsfeld von Gesellschaft, Staat, Profession und Patienten und Patientinnen entfalteten. Der Ausgangspunkt liegt in der Zeit um 1800, eher skizzenhaft führen die Ausführungen sodann durch die zwei Jahrhunderte bis hin zur Behindertenrechtskonvention ins 21. Jahrhundert. Es können nur wenige zentrale Entwicklungen angesprochen werden, die im Hinblick auf ethische Herausforderungen durchleuchtet werden.

## Das psychiatrische Feld um 1800: Humanität, Paternalismus und Ordnungsregimes

Die allgemeinen Menschenrechte erhielten mit der Aufklärung eine deutliche Konturierung und politisch kodifizierte Verbreitung, wenngleich sie – wie allgemein bekannt – keineswegs sogleich auch auf alle Menschen übertragen wurden. Doch fanden sie bald auf Personen Anwendung, die psychisch anders, als sozial störend empfunden wurden und nun als krank galten. Der zentrale Zielbegriff, nach dem dieses Verhältnis gestaltet werden sollte, war der der Humanität. Bis etwa in die zweite Hälfte des 19. Jahrhunderts bestimmte er maßgeblich die Betrachtungen verschiedener psychiatrisch tätiger Ärzte. Was aber sich konkret damit verband, war sehr unterschiedlich.

Untrennbar ist dieser Begriff mit dem Mythos der Befreiung der Irren von den Ketten verbunden, was Philippe Pinel zugeschrieben wird. Wenn auch Pinel eher zögerlich agierte und mehr sein Mitarbeiter Jean Baptiste Pussin die Aktionen förderte [17], ist festzuhalten, dass Pinel und sein Nachfolger Jean Dominique Esquirol ein Konzept entwickelten, welches das Anstaltsleben als wesentlichen Bestandteil der psychiatrischen Therapie auffasste. Die als das Wesen des Irreseins angenommene Entfremdung der Kranken von sich selbst sollte durch ein besonderes Ordnungsregime, das durch und in der Anstalt gelebt wurde, aufgehoben werden.

Philippe Pinel, ab 1793 Leiter des Bicetre und ab 1795 leitender Arzt im Pariser Asyl für weibliche Geisteskranke, der Salpétrière, führte u. a. auf der Basis der vergleichenden Beobachtung eine neue Klassifikation der Geisteskrankheiten ein und entwickelte das „traitement moral“, eine psychische Therapie der Kranken. Diese sog. moralische Behandlung betrachtete die Kranken als nicht ihres Verstandes ganz beraubt, sie galten zugänglich für die Motive der Furcht, der Hoffnung und des Ehrgefühls. Einen glücklichen Erfolg der Therapie erreiche man durch Geschicklichkeit, dagegen verschlimmere üble Behandlung oder gewaltsame Bändigung das Übel und mache es oft unheilbar [11, S. 106]. Es gehe darum, auch Trost zu sprechen, Hoffnung zur Wiederherstellung der Angelegenheiten der Kranken zu erwecken.

Wenn man einen Kranken aber doch auch einmal bändigen müsse, sei es allerdings gestattet, ihn mit einer List zu betrügen und ihm etwas vorzugaukeln [11, S. 102–103]. Sei er dann abgelenkt, könnte eine Übermacht an Wärtern ihn unbemerkt erfassen, ihn in die Höhe heben und in seine Kammer tragen. So könne die Ordnung der Anstalt ohne Gewalttätigkeit erhalten werden. Im Unterschied zu der barbarischen Praxis, nach der Kranke niedergeworfen und Knie auf deren Brust gesetzt würden, werde die kranke Person nun von den Wärtern erhoben und getragen.

Eine bis ins Detail ausgefeilte alltägliche Ordnung der Anstalt sollte das Leben der Insassen regulieren. Nur durch das lebendige Erfahren und eine Verinnerlichung dieser Ordnung sei eine Heilung, also Einpassung in die soziale Ordnung der Gesellschaft, wieder möglich. Die soziale Ordnung der jeweiligen Gesellschaften war eine im Hintergrund wirkende Referenz. In diesen Anstalten war die Macht des Psychiaters allgegenwärtig und wurde von den zahlreichen Wärtern sowohl demonstriert als auch durchgesetzt. Der Umgang mit den Kranken sollte zwar von körperlicher Gewalt möglichst frei gehalten, konnte aber durch kleine Listen und Vorgaukeleien gesteuert werden.

Dieses Ordnungsregime hat in der Folgezeit auch in der Diskussion in Deutschland verschiedene Variationen erfahren. Johann Christian Reil, der Schöpfer des Wortes „Psychiaterie“ und derjenige, der als erster einen Entwurf für das akademische Fach lieferte, formulierte 1803 eine heftige Anklage gegen die Gesellschaft im Umgang mit den Irren. Das Verhalten gegenüber den Unglücklichsten unserer Mitbrüder, so Reil, sei keineswegs der Vernunft gemäß. Gegenüber den Irrenden habe Milde zu walten.

„Wir sperren diese unglücklichen Geschöpfe gleich Verbrechern in Tollkoben, ausgestorbne Gefängnisse, […] und lassen sie daselbst, angeschmiedet an Ketten, in ihrem eigenen Unrath verfaulen. […] sie […] harren des nahen Grabes, das ihren Jammer und unsre Schande zudeckt. […] Die Officianten sind meistens gefühllose, pflichtvergessene, oder barbarische Menschen, die selten in der Kunst, Irrende zu lenken, über den Zirkel hinausgetreten, den sie mit ihrem Prügel beschreiben. […] Die ganze Verfassung dieser tollen Tollhäuser entspricht nicht dem Zweck der erträglichsten Aufbewahrung; und noch weniger der Heilung der Irrenden. […] Wo sind die Früchte unserer gerühmten Cultur, Menschenliebe, Gemeingeist, ächter Bürgersinn […], wenn es auf Rettung Anderer ankommt?“ [13, S. 14–16].

Dieser beeindruckende Standpunkt wurde allerdings durch seine konkreten Vorschläge, die freilich von keiner Praxis im Umgang mit den Kranken getrübt waren, etwas kontrastiert. Für ihn war Irresein dadurch gegeben, dass sich die im Nervensystem vorhandenen Ganglien, die normalerweise mit dem Gehirn verbunden sind, verselbständigen und vom Gehirn unabhängig werden. Irresein bedeutete körperliche Krankheit, bei der die Synthese im Bewusstsein verloren gehe und die Seele von ihrem Standpunkt weggerückt sei. Mit seinem Konzept der psychischen Curmethode sollte mit psychischen Mitteln auf die Seele des Menschen und ihre Vermögen eingewirkt werden, um Heilung zu erreichen. Diese psychischen Mittel sollten die Seele über das Gemeingefühl und die Sinnesorgane, z. B. mithilfe der Sprache oder einer Pantomime, erreichen. Aber anders als der Begriff suggeriert, gehören zu den psychischen Mitteln auch manifeste Einwirkungen auf den Köper, um eben z. B. Sinnesorgane zu aktivieren, wie die Erzeugung von Schmerz und das Provozieren von Ekel und Abscheu etc.. Dabei ging es darum, den „Kranken von der untersten Stufe der Sinnlosigkeit, durch eine Kette von Seelenreizen, aufwärts zum vollen Vernunftgebrauch“ zu „gängeln.“ [13, S. 253] Selbst bei einem von tiefen humanitären Vorstellungen geprägten Menschen war aber von Autonomie, Selbstbestimmung und auch Würde der Irren nicht die Rede. Bei der Herstellung der richtigen Verknüpfung der Ideen und der Wiederherstellung der Willenskraft durfte im Namen der Vernunft auch Zwang, Nötigung, Strafe und Schmerz den unmündigen Kranken zugefügt werden. In diesem Konzept des Zwangs zur Vernunft werden sehr konkrete Auswirkungen der Dialektik der Aufklärung greifbar.

## Menschenrechte und Herausforderungen der somatisch-physiologische Forschungen

Die vielfältigen disziplinierenden, gewalttätigen und restriktiven Maßnahmen innerhalb der Anstalten provozierten nicht selten Gegengewalt und den Wunsch zu flüchten. Abgesehen davon entsprachen sie auch in vielen Fällen nicht dem Selbstbild der Ärzte als humane Helfer. In den 1840er-Jahren kam es in England zu einem ersten die psychiatrische Landschaft in Europa stark beschäftigenden neuen Konzept, dem sogenannten Non-Restraint. Der Umgang mit den Geisteskranken in den Anstalten sollte frei sein von den bis dato immer wieder angewandten Gewaltmaßnahmen wie Zwangsjacken, Zwangsstühlen, Fesselungen und Ähnlichem. In England wurde in den 1830er-Jahren von Robert Gardiner Hill am Lincoln Asylum und von John Conolly am neuen Middlesex County Lunatic Asylum dieses Konzept umgesetzt und alle Arten der mechanischen Beschränkung abgeschafft [12, S. 113–114]. Noch in den 1860er-Jahren begegnete man in Deutschland dieser neuen Behandlungsform mit großer Ablehnung. Eine gewisse Änderung der Haltung zum Non-Restraint trat durch die Entdeckung des Chloralhydrats und dessen Einführung in die psychiatrische Therapie Ende der 1860er-Jahre ein, weil damit nun eine medikamentöse Möglichkeit zur Beruhigung von Tobenden gegeben war.

Alle diese Maßnahmen des Abbaus manifester Gewaltpraktiken in der Psychiatrie förderten auch die Integration des Faches in die Medizin. Damit war aber auch eine verstärkte an den damaligen Methoden der Medizin orientierte Forschung verbunden, was nun wiederum zu neuen ethischen Herausforderungen führte. Wilhelm Griesinger vereinte die hier angesprochenen Tendenzen in sich: Er stand einerseits in der humanitären Tradition und förderte andererseits die wissenschaftliche Profilierung der sich gerade universitär etablierenden Psychiatrie, was er bereits 1845 ausführte. Neben dem Anerkennen des Irreseins als eine Krankheit, war es

„[…] zunächst aber und hauptsächlich der eigentliche Philanthropismus, der den Irren ihre Rechte vom Standpunkte der allgemeinen Menschenrechte vindicirte, […], der es durchsetzte, dass die Gesellschaft in den Irren Menschen anerkannte, denen sie Schutz und Hülfe schuldig ist, dass sie immer mehr Gegenstand zum Zwecke der Heilung angestellter Forschung der Wissenschaft wurden“ [4, S. 341].

Humanität in der Therapie ergebe sich aus der Verbindung von sittlicher Anschauung und theoretischer Einsicht. Da Irresein und psychische Störung aus einer Gehirnaffektion resultieren, fielen sie in die Zuständigkeit des Arztes. In vielen Fällen finde man zwar pathologisch-anatomische Veränderungen wie Entzündungen, Gewebsschwund, doch sei es unmöglich, bestimmte anatomische Veränderungen mit konkreten Krankheitsphänomenen zu korrelieren.

Die bereits von Griesinger angesprochene somatisch-physiologische Forschung machte eine wesentliche Säule der psychiatrischen Erkenntnisgenerierung aus. Die beiden anderen waren die Beobachtung und die klinischen Versuche, wobei es auch zu Überlappungen zwischen den drei Ansätzen kam – wie auch in den anderen medizinischen Disziplinen. Die sich ausweitenden Forschungsaktivitäten waren mit verschiedenen ethischen Herausforderungen verbunden. Dabei trat ein grundsätzliches Problem auf. Im Zusammenhang mit der experimentellen Medizin wies Richard Toellner 1990 auf ein besonderes Dilemma hin, dass er als ethische Aporie bezeichnete. Es sei unethisch, eine Therapie anzuwenden, deren Sicherheit und Wirksamkeit nicht wissenschaftlich geprüft worden ist, denn die Patienten können dabei unnötig zu Schaden kommen. Es sei aber auch unethisch, die Wirksamkeit wissenschaftlich zu prüfen, da bei der Prüfung die Personen geschädigt werden können. Der Konflikt dieser beiden Pflichten ist unaufhebbar. Die Norm ärztlichen Handelns, unter keinen Umständen zu schaden, und die Norm wissenschaftlichen Handelns, Wirksamkeit zu prüfen, schließen sich entweder aus oder schränken sich gegenseitig ein [15]. In ähnlicher Weise hatten bereits 1975 Helmchen und Müller-Oerlinghausen auf das „Paradox“ klinischer Versuche hingewiesen [6]. Da der Konflikt unlösbar ist, versucht man, ihn durch Reglements oder rechtliche Vorgaben etwas zu entschärfen. Diese können zwar das grundsätzliche Problem nicht lösen, aber doch der Selbstbestimmung der betroffenen Personen eine größere Bedeutung einräumen und die medizinischen Experimentatoren zu größerer Wachsamkeit und Sorgfaltsprüfung etc. verpflichten, was zu einer Reduzierung des Risikos beitragen kann.

Solche Regelungen sind gegen Ende des 19. Jahrhunderts verschiedentlich getroffen worden. Dadurch wurde die Selbstbestimmung der Kranken damals deutlich gestärkt. Im Dezember 1900 hieß es in einem Erlass des Preußischen Kultusministeriums, dass medizinische Versuche doch unter allen Umständen ausgeschlossen sind, wenn es sich um eine Person handelt, die noch minderjährig oder aus anderen Gründen nicht vollkommen geschäftsfähig ist, und zudem die betreffende Person nicht ihre Zustimmung zu dem Eingriff in unzweideutiger Weise erklärt hat. Dieser Erklärung hatte eine sachgemäße Belehrung über die aus dem Eingriff möglicherweise hervorgehenden nachtheiligen Folgen vorauszugehen [19, S. 22]. Die Einschränkung der Geschäftsfähigkeit wurde hier explizit als Ausschlusskriterium genannt, d. h. man hat einen freien Willen für die Zustimmung zum Eingriff vorausgesetzt. Nun galten seelische Krankheiten ja als eine Ursache für die Einschränkung des freien Willens. Hier hätte also eine Diskussion über die Zustimmung der psychisch Kranken oder ihrer Vertreter geführt werden müssen. Eine Diskussion dieser Fragen ist aus der Zeit um die Jahrhundertwende in der Psychiatrie nicht bekannt. In den letzten drei Dekaden des 19. Jahrhunderts wurden in der Berliner Gesellschaft für Psychiatrie und Nervenheilkunde immerhin 13 Substanzen hinsichtlich ihrer therapeutischen Einsetzbarkeit an Kranken geprüft. In keinem der Fälle ist in den Publikationen irgendein Hinweis zu finden, dass man die Zustimmung der Kranken oder anderer Personen eingeholt hätte [14].

Hinsichtlich der somatischen Forschung, die psychische Auffälligkeiten mit anatomischen Veränderungen zu korrelieren suchte, entstanden zwei Herausforderungen. Auch wenn bei vielen psychopathologischen Auffälligkeiten keine erklärenden somatischen Befunde gefunden werden konnten, ging man entsprechend dem vorherrschen Paradigma davon aus, dass diese Kausalität irgendwann aufgeklärt sein werde und nahm in vielen Fällen eine besondere individuelle oder familiäre Disposition einer Person für Geisteskrankheiten an. Dies betraf z. B. auch die „conträre Sexualempfindung“ [18]. Die Homosexualität war damit als psychisch krankhafte Störung aus dem moralisch-sittlichen Bewertungssystem der Gesellschaft herausgenommen. Andrerseits aber unterwarf man die betroffenen Personen einer Behandlungsverpflichtung, die über sozialen Druck und andere Mechanismen ihre Wirkung entfaltete und zu teilweise unverantwortlichen Eingriffen in das Selbstbestimmungerecht der betroffenen Menschen führte.

Die zweite Problematik, die mit der somatischen Forschung verbunden ist, betraf die Interpretation bestimmter anatomischer Besonderheiten vor dem Hintergrund von Degenerationstheorie und des Atavismus. Im Zusammenhang mit Mikrozephalengehirnen oder Gehirnen von Kranken mit Imbezillität wurden anatomische Besonderheiten festgestellt, die auch bei Gehirnen von Menschenaffen aufzufinden waren. Damit konnte man Beziehungen zum Tierreich herstellen, wie es z. B. der Genfer Zoologe Karl Vogt in seinem 1866 erschienen Werk „Über Microzephalen oder Affenmenschen“ tat [16]. So wurden in der vergleichenden anatomischen Forschung gewissermaßen Hinweise auf die Existenz „höherer“ und „niederer“ Menschengruppen, die dem Tierreich näherstehen sollen, hergeleitet. Dadurch wuchs die Gefahr, auch psychisch auffällige Personen auf der Grundlage solcher Schemata zu klassifizieren und ihnen eine atavistische Rückentwicklung zu attestieren. Sie wurden so mit dem Nimbus einer dem Tierreich nahestehende Menschenvariante umgeben. In Anbetracht der in der zweiten Hälfte des 19. Jahrhunderts sich verbreitenden Degenerationstheorie, den wachsenden rassischen Abgrenzungsbestrebungen gegenüber „minderwertigen“ und „unzivilisierten“ Völkern und Ethnien eröffneten diese morphologischen Besonderheiten die Möglichkeit, psychisch Kranken mit ihrer Fremdheit nun unter einen gemeinsamen Schirm mit anderen „niedrigstehenden“ und „unzivilisierten“ Ethnien oder sogenannten Rassen zu subsumieren. Zu dem mit dieser Entwicklung im Zusammenhang stehenden Komplex des „kolonialisierten Gehirns“ hat Andreas Heinz vor Kurzem eine Publikation vorgelegt [5].

## Fazit für die Praxis

Die historische Analyse psychiatrischer Entwicklungen in ihrer Verwobenheit mit gesellschaftlichen Faktoren kann die Sichtweise auf Faktoren, die eine aktuelle psychiatrische Praxis bestimmen, sensibilisieren und das Spektrum für mögliche Optionen innovativer psychiatrischer Versorgung erweitern.
